# Research on the secondary branch sizes within crown and primary branch for planted Korean pine in Northeast China

**DOI:** 10.3389/fpls.2025.1548842

**Published:** 2025-08-08

**Authors:** Jiateng Liu, Huilin Gao, Yixi Zou, Qing Hu, Chenyang Zhao

**Affiliations:** College of Forestry, Shenyang Agricultural University, Shenyang, China

**Keywords:** secondary branch model, secondary branch length, secondary branch diameter, mixed-effects model, secondary branch distribution pattern

## Abstract

Branch structure is the fundamental component of an individual tree crown and has an important effect on tree growth and wood quality. A number of studies have focused on the primary branch attributes, but only a few research has been conducted on secondary branch size distribution and models. To analyze the secondary branch structure, we selected 54 Korean pine sample trees from Northeast China and measured a total of 24,053 secondary branches on 717 primary branches. The length and diameter for all the secondary branches and the current-year shoot of the secondary branch distribution were quantified. The allometric relationship between the length and diameter for the primary and secondary branches of the same age and the scaling factor variation within the tree crown were quantified. The nonlinear mixed-effects secondary branch diameter and length model were finally developed. The results indicated that the secondary branches showed an increase in length and diameter, then a decline as they reached deeper into the base of the primary branch. Secondary branch structure varies slightly among trees of varying ages and sizes. The Roeecp function was selected as the basic model to explore the relationship between different variables and the length and diameter of the secondary branches. Absolute distance from the tree tip to the primary branch base and the diameter of the primary branch significantly impacted the branch model, while the individual tree variables and competitive variables had minimal influence on the model. Ultimately, we developed two-level nonlinear mixed-effects models incorporating branch variables for secondary branch length and diameter.

## 1 Introduction

Tree crown is the main place for an individual tree to carry out photosynthesis that are closely related to tree vigor and productivity (Asner et al., 2002; Gill and Biging, 2002; Gao et al., 2023a). Quantitative and morphological characteristics of branches are important determinants of crown structure (Jensen and Long, 1983; Taugourdeau et al., 2012; Dong et al., 2016), since it directly influences light penetration as well as foliage distribution (Liu et al., 2023; Gao et al., 2023b). The crown structure of an individual tree is regarded as an assemblage of primary branches and the secondary branches attached to the primary branches (Remphrey and Powell, 1987; Zheng et al., 2021). The primary branches originating from the stem serve as the fundamental structural units of the crown (Kaitaniemi et al., 2020; Sattler et al., 2014) and determine the crown shape and wood quality (Mäkinen and Hein, 2006; Kershaw et al., 2009; Trincado and Burkhart, 2009). The secondary branch attached to the primary branch was the main place for the long shoots and flowers to grow and was thus the main place for photosynthesis and production accumulation (Taugourdeau et al., 2012; Zheng et al., 2021; Remphrey and Powell, 1987). Therefore, understanding secondary branch development can provide a deeper insight into the detailed crown structure and thus enhance the overall fitness of the crown (Goulet et al., 2000; Chen et al., 2010).

Several studies have demonstrated the hierarchical annual shoot growth existing in some woody plants to improve the efficiency of space acquisition (Suzuki and Suzuki, 2009). Generally, the annual shoot length growth decreased with the increasing branching order of the individual tree emphasizing a pronounced internal control by plant hormones or growth regulators, i.e., a primary branch (i.e., the first order) grew more than a secondary branch (the second order), and so the tertiary and fourth branches (Li et al., 2020; Suzuki, 2003; Kozlowski and Ward, 1961). Despite this fact, more than 60% of the flowers of larch trees originated from the primary and secondary branches following the study of Sieber and Luscher (1995). Thus, several researchers have focused on the characteristics of secondary branches. The length and diameter of the secondary branches are essentially important to reflect the branch status and space utilization (Hein et al., 2008). Thus, the purpose of this study mainly focused on the distribution pattern of the length and diameter of secondary branches. The length and diameter of the primary branch could be quantitatively described by terrestrial laser scanning, but it is almost impossible to the secondary and tertiary branch (Li et al., 2020). Therefore, to describe the detailed information for the secondary branch, it is essentially important to conduct field measurement for the length and diameter of the secondary branches. Developing the allometric relationship between the primary and secondary branches is useful for describing the hierarchical shoot growth of the crown structure (i.e., the length of the annual shoot decreases with increasing branching order). However, this allometric relationship is still unclear, especially for the Korean pine plantation.

Miao et al. (2021b) modeled the number of primary and secondary branches of Korean pine using the mixed-effects approach. Suzuki and Suzuki (2009) conducted an analysis on the current increment of branches of different orders of *Cleyera japonica*. Chen and Sumida (2017) found that both shoot production and death of secondary branches exhibited apparent responses to the ambient light conditions. Moreover, the growth and death of secondary branches also affect the vigor of the primary branches (Chen and Sumida, 2018). However, the specific characteristics for the distribution of the secondary branches within the primary branch and crown, and the information related to the relationship between the distribution pattern and tree age, are still limited. The Richards, Weibull, and Hossfeld equations have been widely used in primary branch length and diameter model development (Dong et al., 2016; Gao et al., 2022; Tong et al., 2025). However, the secondary branch length and branch diameter model have not been developed at present. Selecting appropriate explanatory variables is important for developing an accurate branch attribute prediction model (Colin and Houllier, 1992; Mäkinen and Colin, 1998). As the important component of the crown structure, branch structure is mainly determined by a combination effect of genetic control and external environment (Cushman and MaChado, 2020; Liu et al., 2025). The light condition within the crown varied among the primary branches and was significantly heterogeneous among the secondary branches located at different positions within the primary branches and the entire crown (Taugourdeau et al., 2012). In previous studies on developing the primary branch length and diameter models, the absolute distance from tree the tip to the branch base is a frequently independent variable (Loubère et al., 2004). In addition, tree and stand variables, including total tree height, diameter at breast height, and forest density, were also found to have a significant effect on primary branch length and diameter (Beaulieu et al., 2011; Dong et al., 2016). Competition is a crucial factor affecting the crown structure of individual trees, and related studies have shown that it significantly impacts the size of primary branches (Gao et al., 2022; Tong et al., 2024; Zou et al.,2025). However, the variables affecting the distribution of the length and diameter of the secondary branches are still unclear. Due to the hierarchical structure of the secondary branch data (i.e., the secondary branch nested within the primary branch, the primary branch nested within the individual tree, and the individual tree nested within the sample plot), mixed-effects modeling approach could be used in the length and diameter development of the secondary branches (Pinheiro and Bates, 2000; Bohora and Cao, 2014; Dong et al., 2015).

Korean pine, as a widely planted coniferous tree species in Northeast China, has been used for sawn wood, particleboard, and telecommunication poles (Zu et al., 2011; Liang et al., 2018). The area and volume of Korean pine account for approximately 5.2% and 6.8% of the total in Northeast China, respectively (State Forestry and Grassland Administration, 2021). By our primary analysis, approximately 82% of foliage biomass grows from the secondary branches of the Korean pine in Northeast China. Therefore, quantifying the distribution of the length and diameter of the secondary branch for the planted Korean pine trees is essentially important to evaluate the productivity accumulation and allocation among the different organs, and thus strengthen the understanding of the relationship between crown structure and stem growth for the Korean pine trees. In the present study, we measured the branch length and branch diameter for a total of 24,053 secondary branches in 717 primary branches from 54 sample Korean pine trees in Liaoning province. Based on the large amount of data, we conducted an analysis for (1) quantifying the distribution of the length and diameter of all the secondary branches and only for the current-year shoot of the secondary branch; (2) determining the allometric relationship between the length and diameter for the primary branch and the secondary branch of the same age, and quantifying the allometric relationship variation within the tree crown; and (3) developing the nonlinear mixed-effects secondary branch diameter and length model.

## 2 Materials and methods

### 2.1 Study site

The study was conducted in the Qinghecheng experimental forest farm (124°05′–124°23′E, 41°21′–41°48′N) in Benxi County, Liaoning Province, Northeast China. Dominated by alfisol forest soil, this forest farm is characterized by a terrain of low mountains and hills, with elevations ranging from 400 to 700 m above sea level. It has a temperate continental monsoon climate characterized by hot and rainy summers, and long and dry winters. The annual mean temperature is 6.7°C, with extreme maximum and minimum temperatures of 37.8°C and −37.9°C, respectively. Total annual precipitation ranges from 700 to 900 mm, and the mean annual relative humidity is 67%. Korean pine is the predominant tree species accounting for approximately one-fifth of the total area in this forest farm. Other main tree species in this forest farm include *Larix kaempferi*, *Larix olgensis*, *Pinus sylvestris*, *Quercus mongolica*, *Betula platyphylla*, and *Fraxinus mandschurica*.

### 2.2 Data collection

In Qingcheng experimental forest farm, a total of six forest stands with ages of 28–70 years were selected in 2023. Descriptive statistics for the attributes of the stands are shown in **Table 1**. We conducted a comprehensive survey of the stand conditions and developed three permanent sample plots, each measuring 0.06 ha (20 m × 30 m). These plots were positioned across different slope aspects and positions to ensure a comprehensive reflection of the overall forest stand condition. The diameter at breast height (DBH, cm), total tree height (HT, m), and height to the first living branch (HBLC, m) for all the individual trees from each plot were measured. In addition, the crown width (CW, m), derived from the north, east, south, and west sides, were measured. All individual trees in each plot were sorted by DBH in descending order, and the cumulative basal areas at the breast height for all trees were calculated. The mean values of the DBH for the six trees with the largest DBH and six trees with the smallest DBH from each plot were calculated and recognized as indicating the dominant tree and suppressed tree, respectively. In addition, the trees with a size similar to the quadratic mean diameter for the sample plot were recognized as the intermediate tree. To ensure that the sample plots remain intact for the subsequent measurements, one dominant tree, one intermediate tree, and one suppressed tree were selected outside the permanent sample plot where the site condition is similar to that of the plot. Thus, a total of 54 trees, including 18 dominant, 18 intermediate, and 18 suppressed trees, were selected. DBH, HT, HBLC, and CW in the four cardinal directions for all the sample trees were measured and recorded. The descriptive statistics for the attributes of the sample trees are shown in **Table 2**.

**Table 1:** Symbols, descriptions, and summary statistics for the attributes of the forest stands from the Korean pine plantation in Qingcheng experimental forest farm in Liaoning province, Northeast China.

Level	Variable	Description	Min	Mean	Max	Std
Forest stand (n = 6)	Age (years)	Stand age	28	50	70	14
	D_g_ (cm)	Average diameter at breast height	6.26	28.08	34.87	6.26
	H_dom_ (m)	Average dominant height	12.94	19.16	25.48	3.95
	N (tree/ha)	Density of trees	400	630	1300	309

**Table 2:** Symbols, descriptions, and summary statistics for the attributes of the sample trees in this study.

Level	Variable	Description	Min	Mean	Max	Std
Sample tree (n = 54)	DBH (cm)	Diameter at breast height	7.30	28.23	44.60	8.34
	HT (m)	Tree height	6.72	17.70	24.92	4.35
	CW (m)	Crown width	1.27	2.45	3.98	0.62
	HBLC (m)	Height-to-crown base	1.34	8.77	15.69	4.07
	HD	Height-diameter ratio	0.41	0.65	1.09	0.12

**Figure 1:**
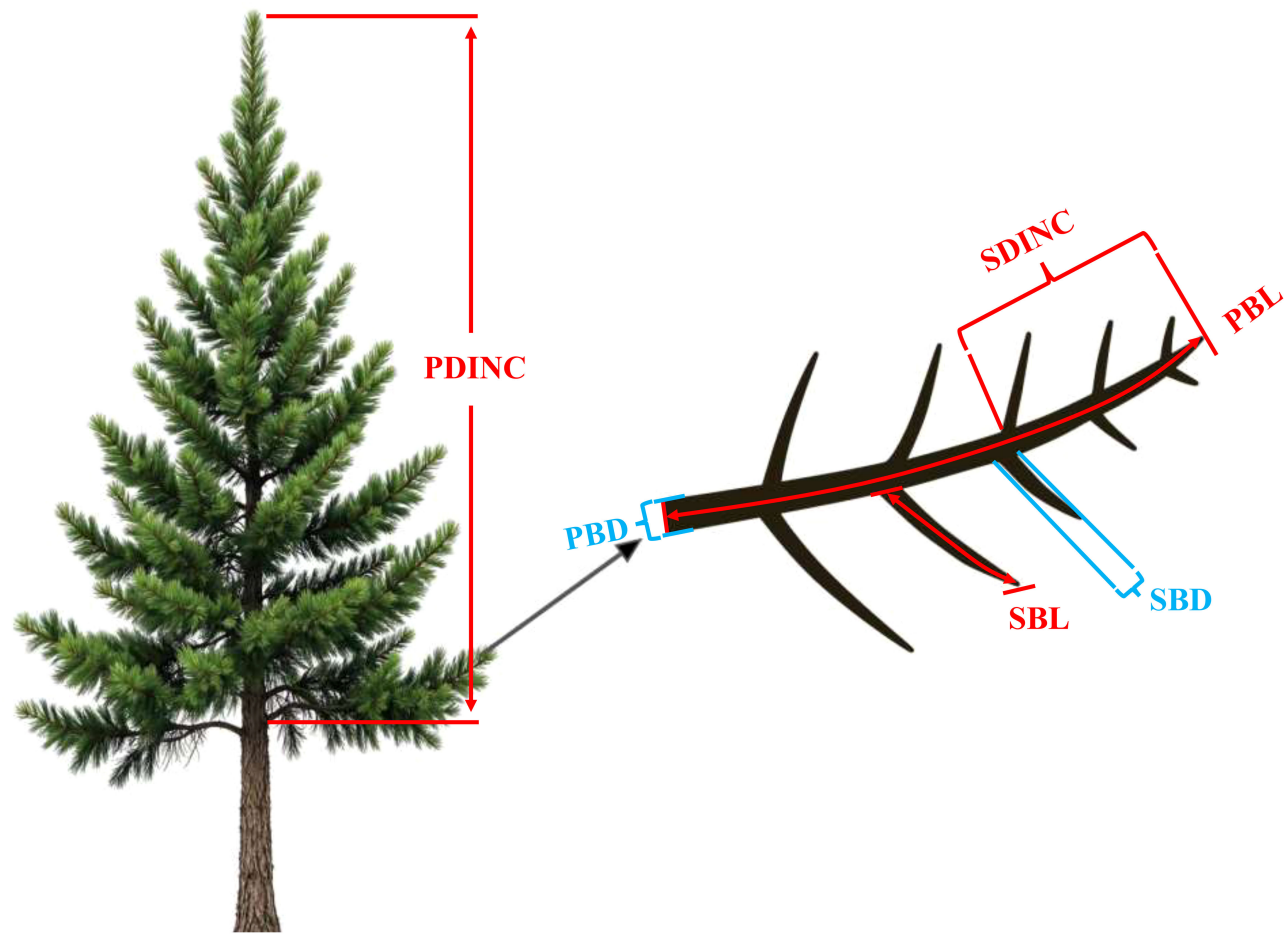
An illustrative description of the variables of the primary branch and secondary branch. PDINC is the distance from the tree tip to the branch base, SDINC is the distance from the top of the primary branch to the base of the secondary branch, PBD is the primary branch diameter, PBL is the primary branch length, SBD is the secondary branch diameter, and SBL is the secondary branch length.

All the sample trees were carefully felled to measure the attributes of the primary and secondary branches. In this study, a whorl containing at least one live branch that remained continuous with the previous whorl was defined as the crown base. The length from the tree tip to the crown base was defined as the crown length (CL, m), and the ratio of CL to HT was defined as the crown ratio (CR). The ratio of HT to DBH was defined as the height–diameter ratio (HD). After completing the measurements, the tree stem was cut into 1-m sections from the stump up, and the section measuring less than 1 m was defined as the tree tip. From the tree tip, all the sections were placed vertically on the ground to ensure that all the branches were maintained in their natural state. The age of the primary branches (PAGE, year) was determined by the growth traces of the trunk at the branch base. For all the living primary branches, branch length (PBL, cm), branch diameter (PBD, mm), branch azimuth (PAZ, °), branch angle (PVA, °), branch chord length (PBC, cm) defined as the distance between branch base and branch tip, and the absolute distance from the tree tip to the primary branch base (PDINC, cm) were measured. In addition, a healthy primary branch indicating the average state of each whorl was selected for most of the whorls as the sample primary branch. For all the sample primary branches, the length (SBL, cm) and diameter (SBD, mm) for all the secondary branches were measured. The absolute distances from the primary branch top to the base of the secondary branch (SDINC, cm) were measured, and the age of all the secondary branches (SAGE, year) were determined by the growth trace of the trunk at the secondary branch base. Notably, in some whorls with fewer than two branches near the base of the crown, the branches are typically either exceptionally large or small. This variation makes it difficult to select sample branches that accurately reflect the average conditions. Consequently, a sample primary branch was not selected in such cases. Finally, we selected 717 sample primary branches and measured a total of 24,053 secondary branches. An illustrative figure for the primary branch and secondary branch are shown in **Figure 1**. The descriptive statistics of the attributes of the sample primary branches and secondary branches are shown in **Table 3**.

**Table 3:** Symbols, descriptions, and summary statistics of the branch attribute variables for the primary branches and secondary branches.

Level	Variable	Description	Min	Mean	Max	Std
Primary branch (n = 717)	PAGE (years)	Branch age	2	11	34	6.52
	PBL (cm)	Branch length	8.00	244.14	661.00	144.77
	PBD (mm)	Branch diameter	4.32	29.81	75.01	14.49
	PBC (cm)	Branch chord length	7.00	217.27	640.00	127.23
	PAZ (degree/°)	Horizontal azimuth angle	0	179	355	104.98
	PVA (degree/°)	Branch insertion angle	0	60	110	13.50
Secondary branch (n = 24,053)	SAGE (years)	Branch age	1	7	32	4.38
	SBL (cm)	Branch length	0.50	25.91	218.00	23.38
	SBD (mm)	Branch diameter	0.40	5.71	37.76	2.59

### 2.3 Data analyses

The mean length and diameter of the secondary branches from each primary sample branch were calculated, respectively. We classified the age of each stand as an age group because the selected stands are even aged. Each stand consists of nine sample trees, and there are a total of six stands. The age groups are as follows: 28, 40, 46, 53, 64, and 70 years. Then, the distribution of the mean secondary branch length and diameter within the entire crown for each age group was analyzed. The response for this distribution pattern to the tree age was determined. The mean secondary branch length and diameter for each whorl of the secondary branch were further calculated for each sample primary branch. The distribution for the mean secondary branch length and diameter within the primary branch were further quantified. In addition, the variation within the entire crown for the distribution pattern for the secondary branch within the primary branch was also determined. The relationship between the primary branch age and current-year shoot length and diameter of the primary branch from the sample trees of different ages.

The power law equation was used to test the allometric relationship between the length and diameter for the primary branch and for the secondary branch of the same age shown as Equations 1, 2.


(1) $$\text{PBL}={\text{a}}_{BL}\cdot SBL^{b_{BL}}$$

(2) $$\text{PBD}={\text{a}}_{BD}\cdot SBD^{b_{BD}}$$

where ${\text{a}}_{BL}$ and ${\text{a}}_{BD}$ are the normalized constants for the allometric relationship for the branch length equation, $b_{BL}$ and $b_{BD}$ are the scaling exponent parameters. The power law equation was transformed into the log version for the purpose of stabilizing the variance as shown in Equations 3 and 4.


(3) $$\log\left(\text{PBL}\right)=\gamma_{BL}+{\text{b}}_{BL}\cdot \text{log}\left(\text{SBL}\right)$$

(4) $$\log\left(\text{PBD}\right)=\gamma_{BD}+{\text{b}}_{BD}\cdot \text{log}\left(\text{SBD}\right)$$

where $\gamma_{BL}$ and $\gamma_{BD}$ are log( ${\text{a}}_{BL}$ ) and log( ${\text{a}}_{BD}$ ). Model 3 was used to fit the allometric relationship between the secondary branch length and the primary branch length. Similarly, Model 4 was used to fit the allometric relationship between the secondary branch diameter and the primary branch diameter. For both of the branch length and diameter, the allometric relationships at the three levels were studied. For the primary branches of the same age, the allometric relationships for the length and diameter between the primary branch and the secondary branch attached to the primary branch base were studied. The scaling exponent parameters for the primary branches with the different ages were calculated. For the secondary branches of the same age, the allometric relationship for the length between the secondary branch and the length from the primary branch tip downward to the location where the same secondary branch attached, and for the diameter between the secondary branch and the location at the primary branches where the same secondary branch was attached, were studied. The scaling exponent parameters for the secondary branches of different ages were calculated. For the trees of the same age, the allometric relationship between the length and diameter of the primary branch and secondary branch attached to the primary branch base was calculated, and scaling exponent parameters were calculated for trees of different ages. Ordinary least square was used to estimate the parameters. Bootstrap percentile method (He et al., 2024) was used to test the significant difference in the scaling parameters of ${\text{b}}_{BL}$ and ${\text{b}}_{BD}$ for each whorl for the primary branch and for the secondary branch. A total of 3,000 bootstrap replicates of the model were generated, and the 95% confidence intervals for each parameter were calculated.

### 2.4 Secondary branch length and diameter model development

Based on the preliminary analysis for the distribution of the secondary branch length and branch diameter within the crown, the secondary branch length and diameter models were further developed. Logistic function (Adolt et al., 2012), Richards function (Jia and Chen, 2019), Roeecp function (Sun et al., 2017), Weibull function (Abino et al., 2016), Hossfeld function (Dong et al., 2016), Gompertz function (Parhizkar et al., 2010), Mitscherlich function (Marino et al., 2010), and Korf function (Li et al., 2000), which have been widely used in the primary branch modeling, were also used as the candidate secondary branch length and diameter models. The forms of candidate models are shown in **Table 4**. SDINC was first incorporated into the secondary branch length and diameter models. To further improve the prediction ability, the commonly used tree, stand, and competition variables in the primary branch model were also incorporated into the basic models. The tree variables considered were HT, DBH, HD, CW, CL, and CR, and the branch variables were PBL, PBD, and PDINC. The competition variables that performed excellently in previous studies were also used and compared (Gao et al., 2022; Liu et al., 2024). The relationships between each variable and SBL and SBD were primarily analyzed. The variables that showed effects on the SBL and SBD were incorporated into the models. All the combinations of candidate variables were tried and compared. We used nonlinear least squares regression to fit the basic model, and the candidate model that showed the best performance was selected as the optimal basic model. The specific variable that showed the best contribution to the model was selected to be incorporated into the model. Akaike information criterion (AIC), root mean squared error (RMSE), and the adjusted coefficient of determination (R_a_^2^) were used to evaluate model performance. The significance of the model parameters associated to the variables was also considered.

**Table 4:** The forms and fitting accuracy statistics for secondary branch candidate models.

Models	Function	AIC for SBL models	AIC for SBD models
M1	$Y=a*{\left[1-\text{exp}\left(-b*SDINC\right)\right]}^{c}$	——	108,350
M2	$Y=a*\left[1-\text{exp}\left(-b*SDINC^{c}\right)\right]$	211,270	108,360
M3	Y=a/[1+b∗exp(−c∗SDINC)]	211,343	108,422
M4	$Y=a/\left(1+b*SDINC^{-c}\right)$	——	108,363
M5	Y=a∗exp(−b/SDINC)	211,768	109,327
M6	Y=a∗[1−exp(−b∗SDINC)]	211,271	109,515
**M7**	$Y=a*SDINC^{b}*\text{exp}\left(-c*SDINC\right)$	211,255	108,347
M8	$Y=a*\text{exp}\left(-b*SDINC^{-c}\right)$	——	——
M9	Y=a∗exp[−b∗exp(−c∗SDINC)]	211,277	108,390

Y is the secondary branch length or the secondary branch diameter. SDINC is the absolute distances from the primary branch top to the base of the secondary branch.

Due to the hierarchical structure and spatial interrelation of the branch data, two-level (primary and secondary branch levels) nonlinear mixed-effects models were developed (Pinheiro and Bates, 2000). The nonlinear mixed-effects models with two-level random effects are formulated as follows (Pinheiro and Bates, 2000):


(5) $$\begin{array}{c} y_{ijk}=f\left(\varphi_{ijk},v_{ijk}\right)+e_{ijk},\;i=1,\cdots ,M,\;j=1,\cdots ,M_{i},\;k\\ =1,\cdots n_{ij}, \end{array}$$

(6) $$e_{ijk}∼N\left(0,R_{ij}\right)$$

where *y_ijk_* is the secondary branch length or diameter of the *kth* observation on the *jth* whorl of the secondary branch from the *ith* primary branch; *M* is the number of the primary branch level, *M_i_* is the number of the whorl of the secondary branch from the *ith* primary branch, and *n_ij_* indicates the number of observations on the *jth* whorl of the secondary branch from the *ith* primary branch; *e_ijk_* accounts for within-group variance and correlation, and is assumed to be a normal distribution with zero expectation and a positive-definite variance–covariance structure *R_ij_*; f(·)  is a real-valued and differentiable function of a group-specific parameter vector *φ_ijk_* and a covariate vector *v_ijk_*; and the parameter vector *φ_ijk_* could be defined as follows:


(7) $$\varphi_{ij}=A_{ijk}\beta +B_{ijk}b_{i}+M_{ijk}b_{ij},\;b_{i}∼N\left(0,D_{1}\right),\;b_{ij}∼N\left(0,D_{2}\right)$$

where *β* is the p*-*dimensional fixed-effect parameter vector; *b_i_* and *b_ij_* are the first and second-level random effects, which are independent normally distributed q_1_- and q_2_- dimensional vectors with zero means and variance–covariance matrices *D_1_* and *D_2_*; *b_i_*, *b_ij_*, and *e_ijk_* are mutually independent; and *A_ijk_*, *B_ijk_*, and *M_ijk_* are the corresponding design matrices. *D_1_* and *D_2_*, which are both assumed to be unstructured in this study, explain the variability between the primary branches and the whorl of the secondary branches. Moreover, to clarify the within-group variance and autocorrelation structure of *R_ij_*, it can be expressed as follows:


(8) $$R_{ij}=\sigma^{2}G_{ij}^{0.5}\Gamma_{ij}G_{ij}^{0.5}$$

where *σ^2^* is an error variance, which is a scaling factor for the error dispersion; *G_ij_* is an *n_pt_ × n_pt_* diagonal matrix that is used to describe the within-group heteroscedasticity variances; and *Γ_ij_* is an *n_pt_ × n_pt_* matrix showing the within-group autocorrelations structure of errors. We considered the exponential function and power function to address the issue of heteroskedasticity (Liu et al., 2025). Furthermore, we considered using the autoregressive structure AR (1), the moving average structure MA (1), and the autoregression moving average structure ARMA (1, 1) to effectively model the correlation of within-group errors (Pinheiro and Bates, 2000). For the two levels of random effects, all the parameters or combinations were used as random effects. The AIC and logarithm likelihood values for all possible combinations of random effects were compared. The specific model with the smallest AIC and largest Log-likelihood was selected as the best. The likelihood ratio test (LRT) was performed to prevent overparameterization of the models. The parameter estimations were estimated by the *nlme* package of the R software (Pinheiro et al., 2021; R Core Team, 2021).

## 3 Results

### 3.1 Distribution of secondary branch length and diameter within crown

**Figure 2:**
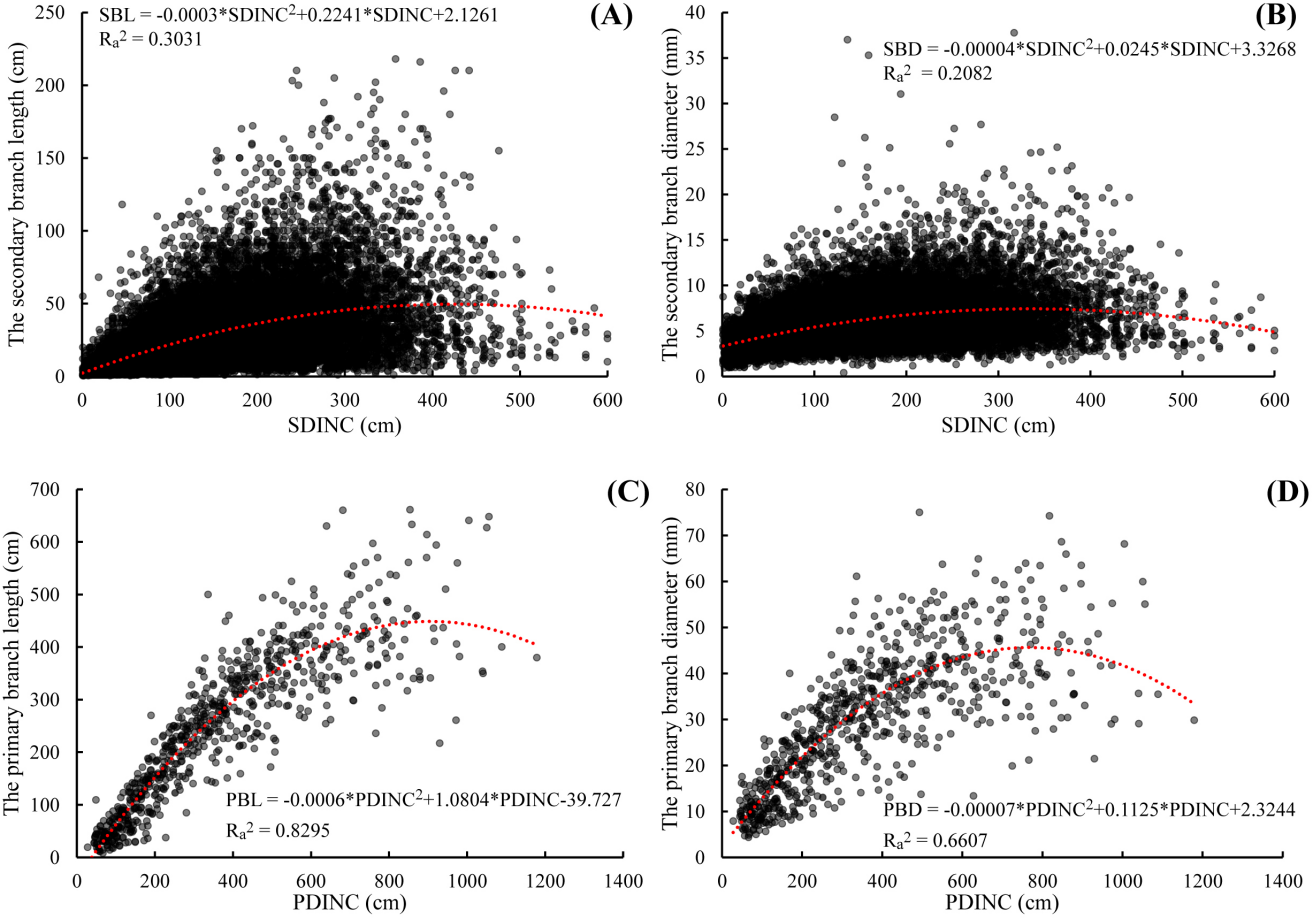
Relationship between the secondary branch length and the SDINC **(A)**, the secondary branch diameter and the SDINC **(B)**, the primary branch length and PDINC **(C)**, and the primary branch diameter and PDINC **(D)**. SDINC is the distance from the top of the primary branch to the base of the secondary branch. PDINC is the distance from the tree tip to the branch base. The same applies below.

As showed in **Figure 2**, both secondary branch length and diameter generally increased first and then decreased slightly from the primary branch tip to the branch base with an average trend. The distribution of the secondary branch length showed a similar pattern to the secondary branch diameter. The variation of the secondary branch length (**Figure 2A**) is larger than that of the branch diameter from the primary branch tip to the branch base (**Figure 2B**). We also found that this distribution pattern is similar to that of the primary branch length (**Figure 2C**) and diameter (**Figure 2D**) within the crown.

**Figure 3:**
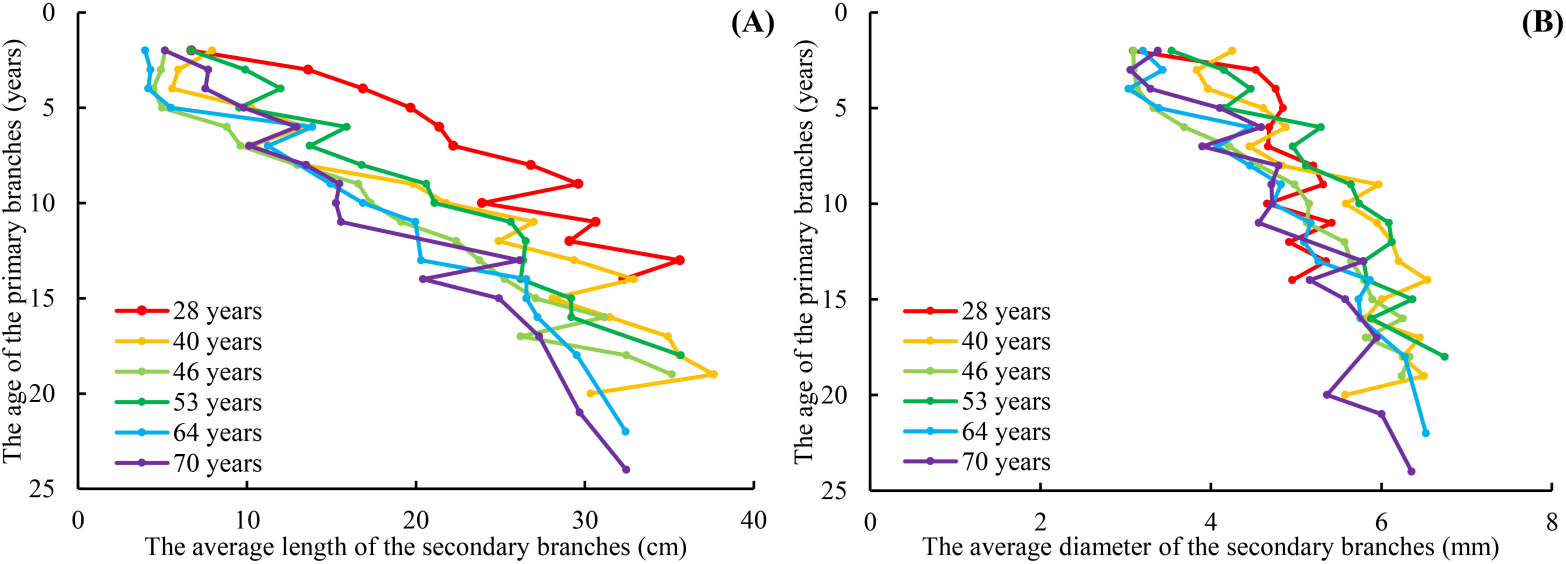
The relationship between the average length of the secondary branches and PAGE **(A)**, and the relationship between the average diameter of the secondary branches and PAGE **(B)**. PAGE is the age of the primary branches. The red line indicates that the age of the planation is 28 years. The orange line indicates that the age of the planation is 40 years. The red line indicates that the age of the planation is 46 years. The bottle green line indicates that the age of the planation is 53 years. The blue line indicates that the age of the planation is 64 years. The purple line indicates that the age of the planation is 70 years. The same applies below.

The range of the mean secondary branch length for the primary branch from the tree top to the crown base is 4.0–37.6 cm. The mean secondary branch length showed an increasing tendency from the tree tip downward to the crown base, and this trend tends to stabilize in the lower part of the crown. Generally, the mean secondary branch length decreased with increasing tree age and expressed as the regularity of 28 > 40 > 53 > 46 > 64 > 70 (**Figure 3A**). The range of the mean secondary branch diameter from the tree top downward to the crown base is 3.03–6.74 mm and also showed a slightly increasing tendency from the tree tip downward to the crown base, but not as apparent as that of the branch length (**Figure 3B**). As for the variations of this distribution between the tree ages, the regularity of the secondary branch diameter from the tree tip downward to the crown base generally decreased with increasing tree age, but did not express the obvious regularity as branch length. As for the trees of 28 years, the mean secondary branch diameter remained nearly constant from the fourth whorl to the crown base.

### 3.2 Distribution of secondary branch length and diameter within the primary branch

**Figure 4:**
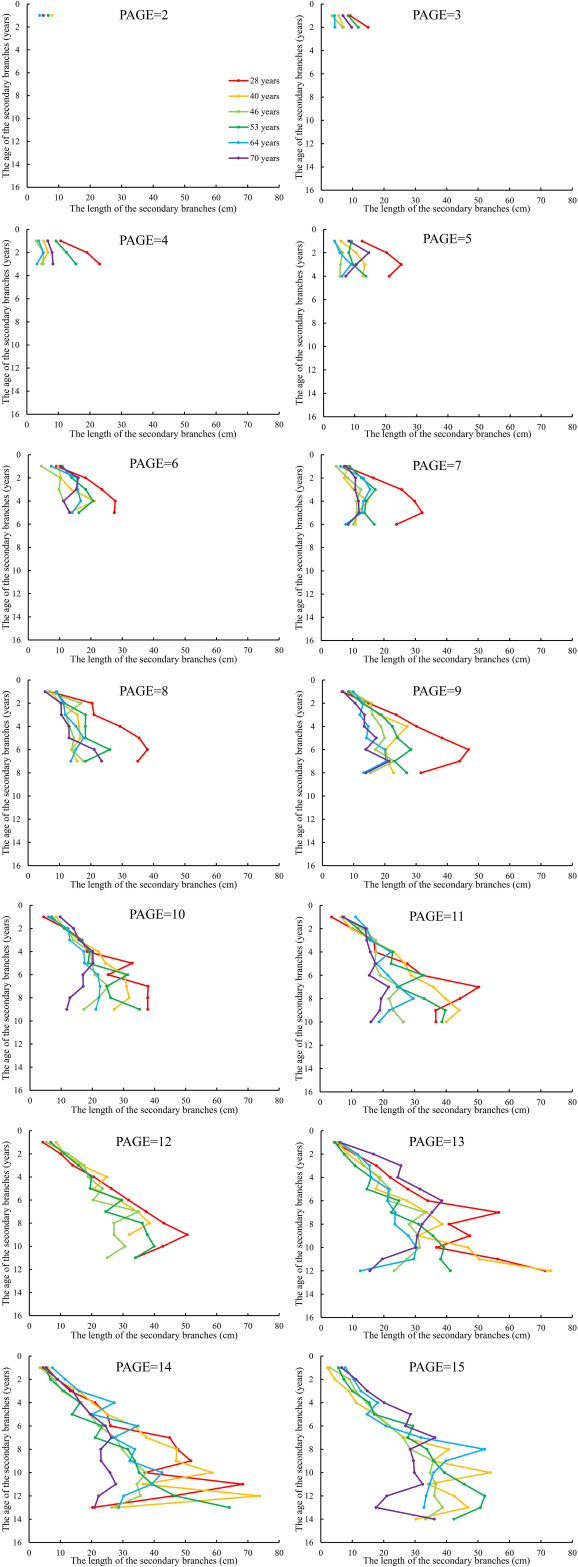
The relationship between the average length of the secondary branches and SAGE. SAGE is the age of the secondary branches. The same applies below.

**Figure 5:**
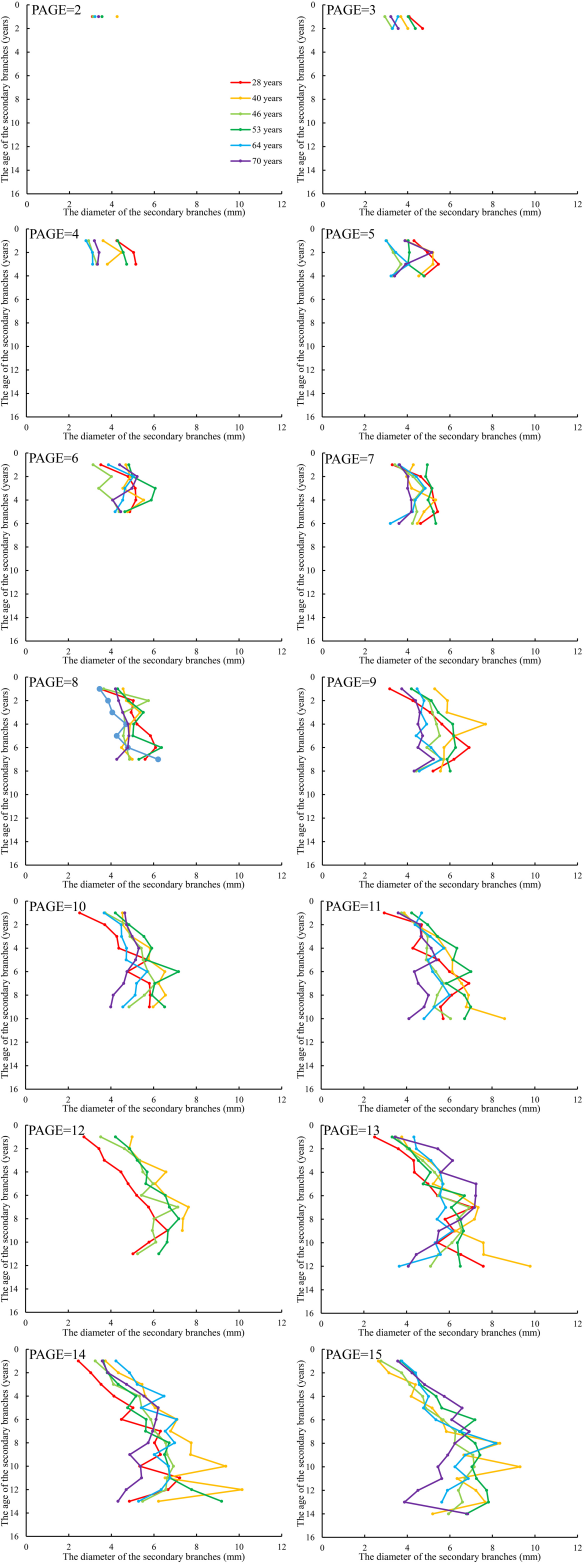
The relationship between the average diameter of the secondary branches and SAGE.

**Figure 6:**
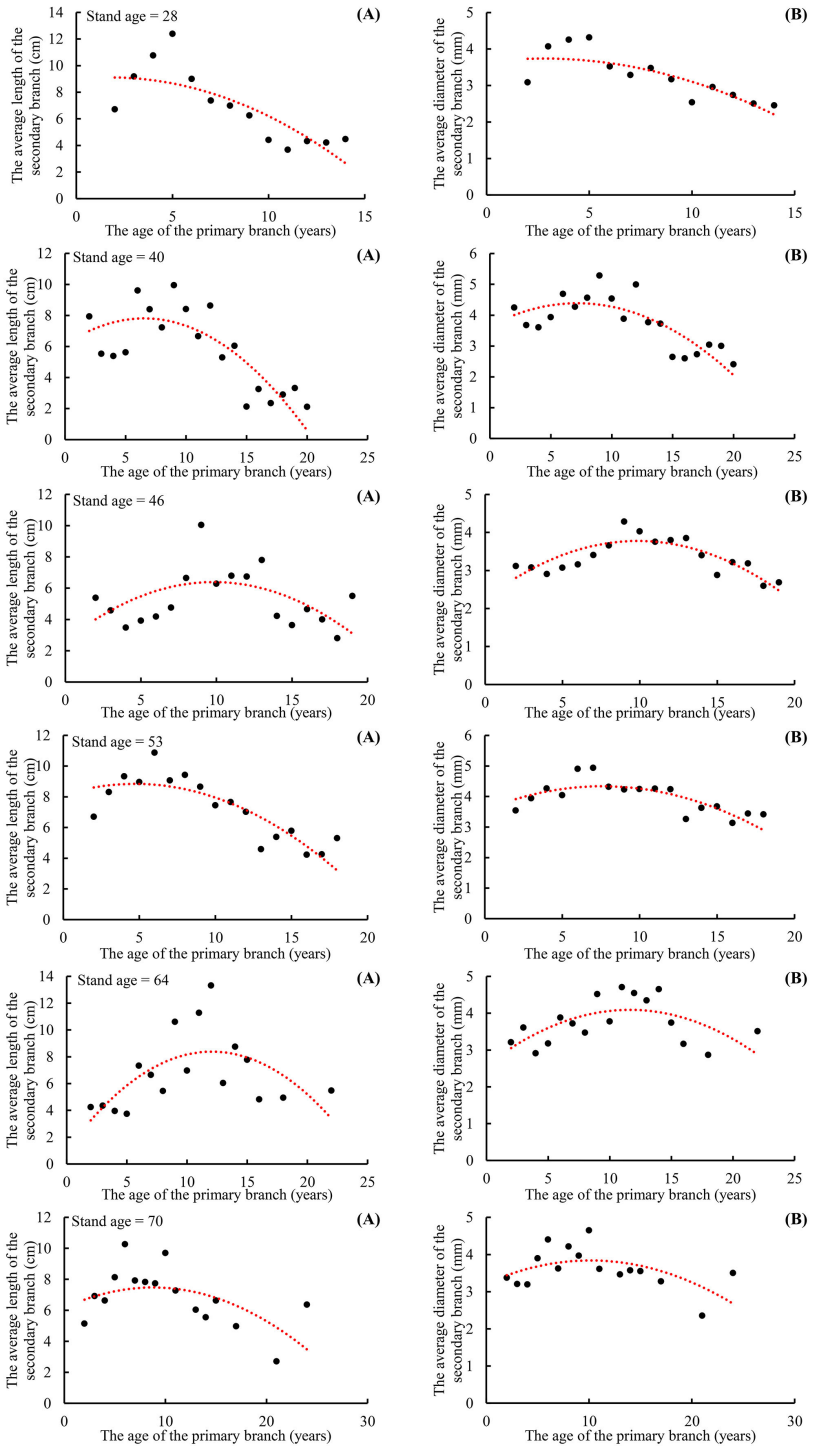
The relationship between the average length of newly germinated secondary branches and PAGE **(A)**, and the relationship between the average diameter of the newly germinated secondary branches and PAGE **(B)**.

We further averaged the branch length and diameter of the secondary branches from each whorl of the primary sample branch from the first whorl to the last whorl, respectively. The mean values of the secondary branch length and diameter of each whorl for each primary sample branch of each of the nine sample trees of the same age were calculated. The differences in the distribution of the secondary branch length and diameter within the primary branch from the tree tip downward to the crown base for different tree ages are shown in **Figures 4** and **5**, respectively. The mean values of the secondary branch length and diameter generally increased and then decreased from the primary branch tip downward to the primary branch base, and the inflection points for the secondary branch length and diameter are 0.78 and 0.76, respectively. As for the primary branch of the same age from individual trees, it was clearly indicated that the mean secondary branch length of each whorl generally showed a decreasing tendency with increasing tree age when the primary branch age was lower than 6 years. When the age of the primary branch was more than 6 years, the mean length of the secondary branches located within the SDINC of 0.22 of the primary branch generally increased with increasing tree age. As a specific case for the trees with 28 years, the mean secondary branch length is significantly longer than that of trees of different ages when PAGE ≤ 8, and the distinction between different ages become insignificant with increasing of PAGE. We further studied the relationship between the primary branch age and the length and diameter of the current-year secondary branches. With increasing primary branch age, the length and diameter of the current-year secondary branches either gradually decreased or first increased and subsequently decreased (**Figure 6**).

### 3.3 Scaling exponent variations within the crown

**Figure 7:**
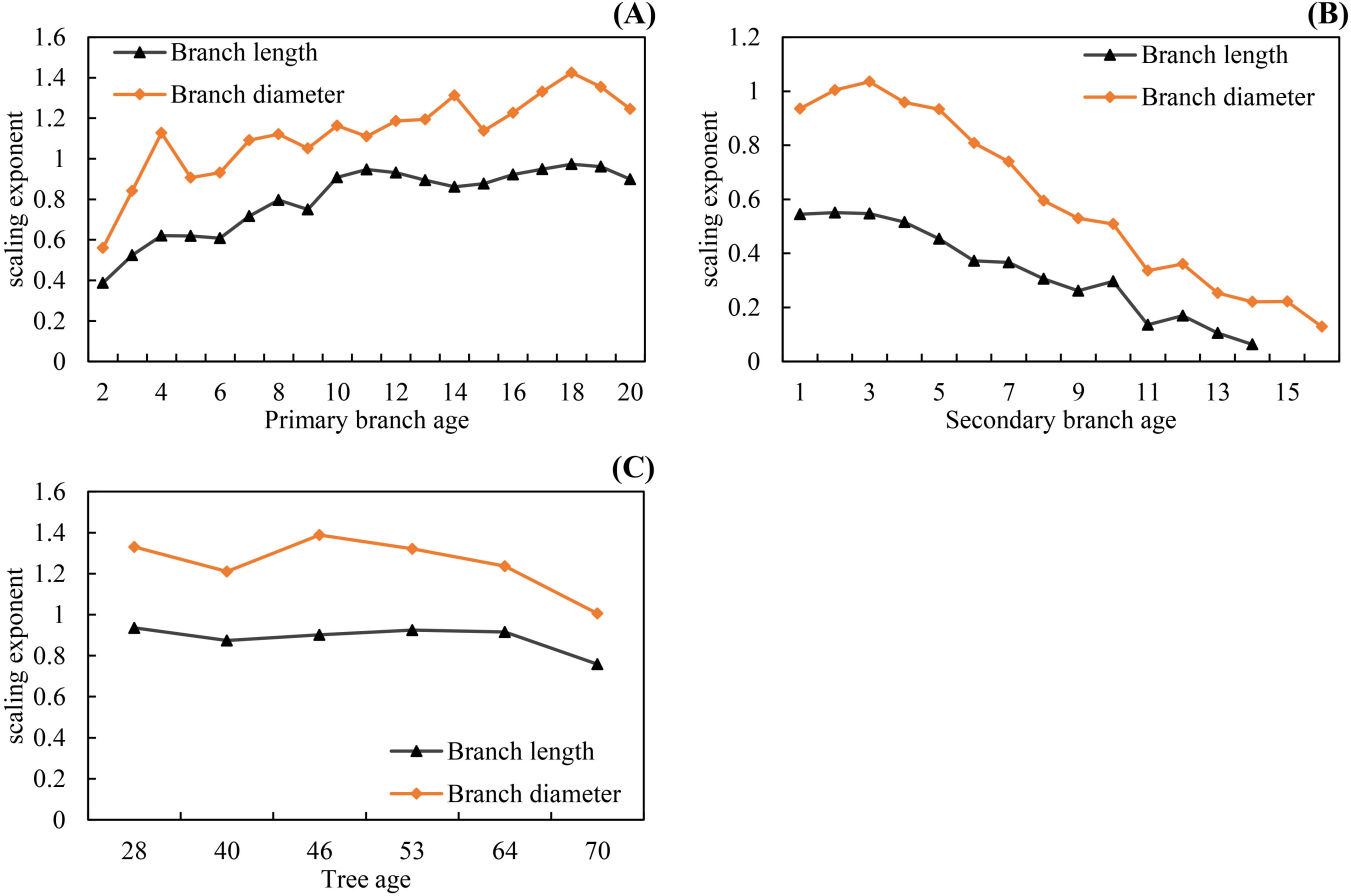
The scaling parameters increased from the tree tip downward to the crown base for both branch length and diameter allometric relationships **(A)**, and decreased from the secondary branch tip downward to the branch base for both branch length and diameter allometric relationships **(B)**, and slightly decreased with increasing tree age for both branch length and diameter allometric relationships **(C)**.

The scaling exponent parameter of the allometric relationship for the branch length ( $\gamma_{BL}$ from Model 3) between the primary and secondary branches ranges from 0.3874 to 0.9733 for the primary branches of different ages (**Figure 7A**), and the allometric relationship for branch diameter ( $\gamma_{BD}$ from Model 4) between the primary and secondary branches ranges from 0.5597 to 1.4247 for the primary branches of different ages (**Figure 7A**). The scaling exponent parameter of the allometric relationship for branch length between the secondary branch and the length from the primary branch tip downward to the location on the primary branch where the same secondary branch was attached ranges from 0.0631 to 0.5508 for the secondary branches of different ages (**Figure 7B**), and the allometric relationship for branch diameter between the secondary branch and the location on the primary branches where the secondary branches were attached ranges from 0.1285 to 1.0352 (**Figure 7B**). The scaling exponent parameter of the allometric relationship for the branch length between the primary and the secondary branches for individual trees of different ages ranges from 0.7584 to 0.9357 (**Figure 7C**), and the allometric relationship for the branch diameter ranges from 1.0054 to 1.3882 (**Figure 7C**).

### 3.4 Secondary branch length and diameter model development

#### 3.4.1 Basic model selection

As shown on **Table 4**, the Roeecp model exhibits the smallest AIC for both secondary branch length and diameter. Finally, the Roeecp model was selected as the basic model for developing the secondary branch length and diameter models. The estimates for the basic secondary branch length and diameter models are provided in **Tables 5** and **6**, respectively. The secondary branch length and diameter models are formulated as follows:

**Table 5:** Parameter estimates and fitting accuracy statistics for the basic model, optimal model, and mixed model of secondary branch length.

Mixed effects model	Parameters	Equation 5		Equation 7		Equation 9	
		Estimates	*P* Value	Estimates	*P* Value	Estimates	*P* Value
Fixed-effects parameters	a	0.364	<0.0001	0.188	<0.0001	0.197	<0.0001
	b	0.928	<0.0001	0.974	<0.0001	0.989	<0.0001
	c	0.002	<0.0001	0.002	<0.0001	0.003	<0.0001
	a_1_			3.0*10^-4^	<0.0001	3.0*10^-4^	<0.0001
Variance parameters	$\sigma^{2}$					251.6	
	$\sigma_{b_{i}}^{2}$					6.2 * 10^−3^	
	$\sigma_{c_{i}}^{2}$					4.0 * 10^−6^	
	$\sigma_{b_{i}c_{i}}$					0.842	
	$\sigma_{b_{ij}}^{2}$					1.9 * 10^−3^	
Evaluation indices	MAE	13.07		12.60		10.42	
	RMSE	19.54		18.96		15.09	
	R_a_^2^	0.302		0.343		0.584	
	AIC	211255		209815		205261	
	LL	-105623		-104902		-102622	


(9) $$\text{SB}{\text{L}}_{\text{ijk}}=\text{a}*\text{SDIN}{\text{C}}_{\text{ijk}}{\;}^{b}*exp\left(-\text{c}*\text{SDIN}{\text{C}}_{\text{ijk}}\right)$$

(10) $$\text{SB}{\text{D}}_{\text{ijk}}=\text{a}*\text{SDIN}{\text{C}}_{\text{ijk}}{\;}^{b}*exp\left(-\text{c}*\text{SDIN}{\text{C}}_{\text{ijk}}\right)$$

where *SBL_ijk_* and *SBD_ijk_* are the secondary branch length (cm) and secondary branch diameter (mm) of measurement *k* for the *jth* whorl of the secondary branch from the primary branch *i*, respectively; *SDINC_ijk_* is the corresponding absolute distance from the top of the *ith* primary branch to the *jth* whorl of the secondary branch; and *a*, *b*, *c* are the parameters to be estimated.

#### 3.4.2 Additional predictor variables

All the variables were incorporated into different positions of the SBL and SBD models, and the fitting accuracy was compared. Finally, the variable PDINC was incorporated into the SBL model (Equation 11), and PBD was incorporated into the SBD model (Equation 12). The fitting accuracy of the models was not improved by introducing other variables. The fitting accuracy of the secondary branch length and diameter models was significantly improved compared to the basic models. In addition, all the parameters of both the SBL and SBD models had good stability. The results of parameter estimation and fitting accuracy for the SBL and SBD models are shown in **Tables 5** and **6**, respectively.

**Table 6:** Parameter estimates and fitting accuracy statistics for the basic model, optimal model, and mixed model of secondary branch diameter.

Mixed effects model	Parameters	Equation 6		Equation 8		Equation 10	
		Estimates	*P* Value	Estimates	*P* Value	Estimates	*P* Value
Fixed-effects parameters	a	1.408	<0.0001	1.292	<0.0001	1.321	<0.0001
	b	0.309	<0.0001	0.276	<0.0001	0.270	<0.0001
	c	4.1*10^-4^	<0.0001	1.2*10^-3^	<0.0001	1.4*10^-3^	<0.0001
	b_1_			1.9*10^-3^	<0.0001	2.2*10^-3^	<0.0001
Variance parameters	$\sigma^{2}$					3.649	
	$\sigma_{b_{i}}^{2}$					2.1 * 10^−3^	
	$\sigma_{c_{i}}^{2}$					1.9 * 10^−6^	
	$\sigma_{b_{i}c_{i}}$					0.891	
	$\sigma_{b_{ij1}}^{2}$					4.1 * 10^−7^	
Evaluation indices	MAE	1.651		1.588		1.330	
	RMSE	2.316		2.236		1.811	
	R_a_^2^	0.209		0.263		0.511	
	AIC	108347		106611		103494	
	LL	-54169		-53300		-51738	


(11) $$\begin{array}{c} \text{SB}{\text{L}}_{\text{ijk}}=\left(\text{a}+{\text{a}}_{1}*\text{PDIN}{\text{C}}_{\text{ijk}}\right)*\text{SDIN}{\text{C}}_{\text{ijk}}{\;}^{b}*exp(\\ -\text{c}*\text{SDIN}{\text{C}}_{\text{ijk}}) \end{array}$$

(12) $$\text{SB}{\text{D}}_{\text{ijk}}=\text{a}*\text{SDIN}{\text{C}}_{\text{ijk}}{\;}^{\left(\text{b}+{\text{b}}_{1*}\text{PB}{\text{D}}_{\text{ijk}}\right)}*exp\left(-\text{c}*\text{SDIN}{\text{C}}_{\text{ijk}}\right)$$

where *PDINC_ijk_* is the absolute distance from the tree tip to the *ith* primary branch base, *PBD_ijk_* is the diameter of the *ith* primary branch; and *a*, *a*_1_ *, b, b*_1_ *, c* are the parameters to be estimated; other variables were defined in the same way as above.

#### 3.4.3 Nonlinear mixed-effects branch models

To explain the hierarchical structure and spatial interrelation of the branch data, two-level mixed effects SBL and SBD models were developed. All the combinations of random effects for Equations 11 and 12, considering both primary branch effects and the nested effects of the secondary branch, were calculated and compared. Fitted to the data, the mixed-effects models did not converge when the number of random effect parameters exceeded four. As for the mixed-effects secondary branch length model of Equation 13, incorporating primary branch effects on parameters *b* and *c*, as well as the secondary branch effects on parameter *b*, yielded the smallest AIC and the largest logarithm likelihood. As for the mixed-effects secondary branch diameter model of Equation 14, incorporating the primary branch effects on parameters *b* and *c*, and the secondary branch effects on parameter *b*_1_, achieved the smallest AIC and the largest logarithm likelihood. Neither of the models reached convergence when dealing with the within-group variance and autocorrelation structure of the error term. The parameter estimates and fitting statistics for the mixed-effects secondary branch length and diameter models are shown in **Tables 5** and **6**, respectively.


(13) $$\begin{array}{c} \text{SB}{\text{L}}_{\text{ijk}}=\left(\text{a}+{\text{a}}_{1}\cdot \text{PDIN}{\text{C}}_{\text{ijk}}\right)\cdot \text{SDIN}{\text{C}}_{\text{ijk}}{\;}^{\left(\text{b}+{\text{b}}_{\text{i}}+{\text{b}}_{\text{ij}}\right)}\\ \cdot \text{exp}\left[-\left(\text{c}+{\text{c}}_{\text{i}}\right)\cdot \text{SDIN}{\text{C}}_{\text{ijk}}\right]+{\text{e}}_{\text{ijk}} \end{array}$$

(14) $$\begin{array}{c} \text{SB}{\text{D}}_{\text{ijk}}=\text{a}\cdot \text{SDIN}{\text{C}}_{\text{ijk}}{\;}^{\left[\text{b}+{\text{b}}_{\text{i}}+({\text{b}}_{1}+{\text{b}}_{\text{ijk}})\cdot \text{PBD}\right]}\\ \cdot \text{exp}\left[-\left(\text{c}+{\text{c}}_{\text{i}}\right)\cdot \text{SDIN}{\text{C}}_{\text{ijk}}\right]+{\text{e}}_{\text{ijk}} \end{array}$$

where *a*, *a*_1_, *b*, *b*_1_, *c* are the fixed-effects parameters, *b_i_* and *c_i_* are the random parameters caused by the *ith* primary branch on *b* and *c*, respectively, and *b_ij_* and *b_ij_*_1_ are the random parameters caused by the *jth* secondary branch nested in the *ith* primary branch on *b* and *b*_1_, respectively.

**Figure 8:**
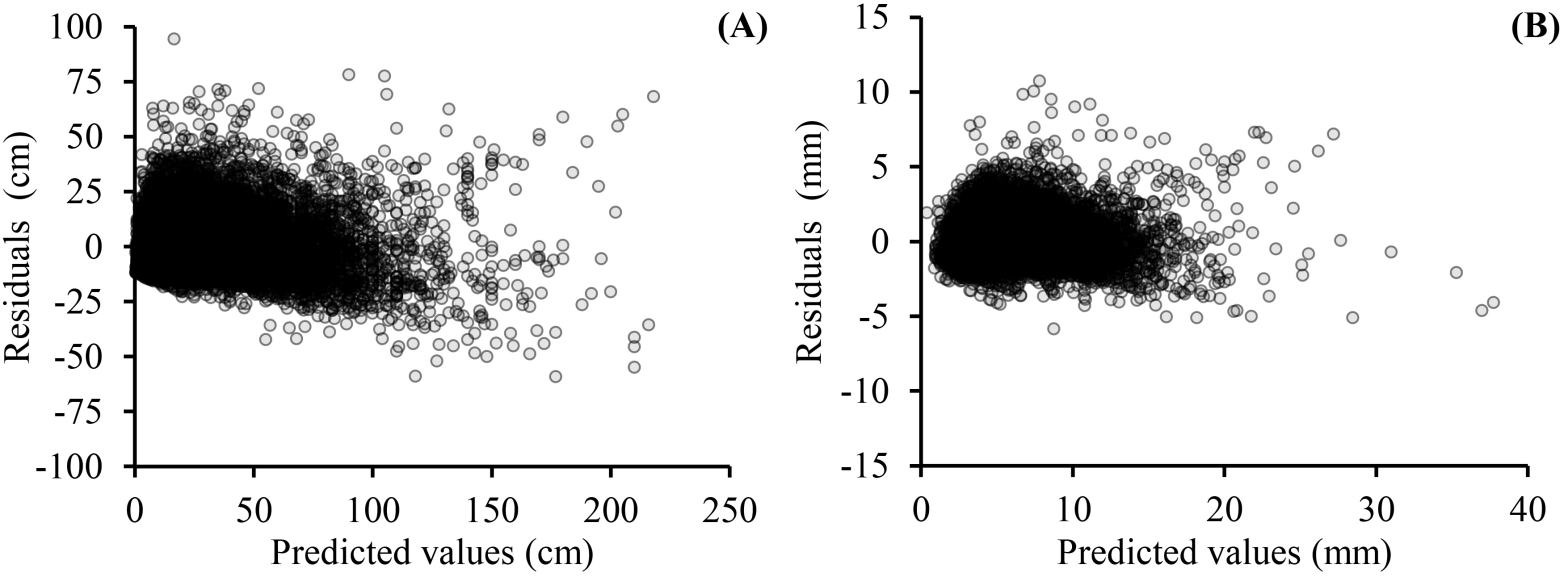
Residual plots of mixed-effects models for secondary branch length **(A)** and secondary branch diameter **(B)**.

**Figure 9:**
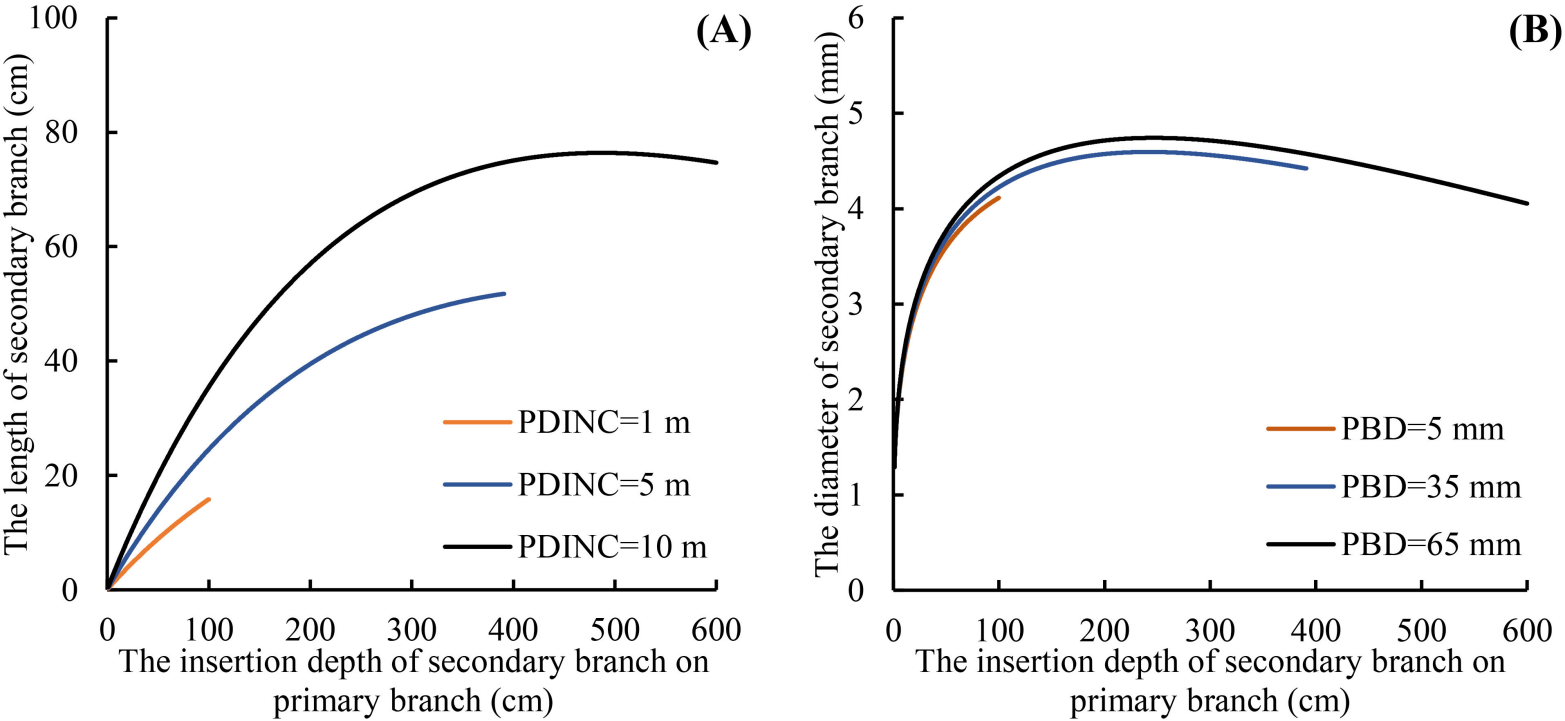
Relationship between the secondary branch length and the SDINC **(A)**, and the relationship between the secondary branch diameter and the SDINC **(B)**. PBD is the primary branch diameter.

As shown on **Tables 5** and **6**, the fitting results of the basic models and the mixed-effects models show that AIC and −2LL were significantly reduced after adding random effects to the basic models. At the same time, the R_a_^2^ of both growth models was improved, and the MAE and RMSE were reduced indicating that the mixed models greatly improved the fitting effects of the secondary branch length and diameter models. Furthermore, the residual plots of the mixed-effects secondary branch length and diameter model performed well than the basic model (**Figure 8**). Based on the parameters of the fixed effects in the mixed models, the relationship between the length and diameter of the secondary branches and SDINC is simulated and predicted (**Figure 9**). Both PDINC and PBD reflect the position and size of the primary branch in the crown and indirectly reflect the primary branch age. As shown in **Figures 9A** and **B**, the relationships of the secondary branch length and diameter with SDINC were consistent with the above description. In addition, there are significant differences in the length of the secondary branches among the different primary branches, but only slight differences in the secondary branch diameter, which also indirectly confirms the above analysis results.

## 4 Discussion

**Figure 10:**
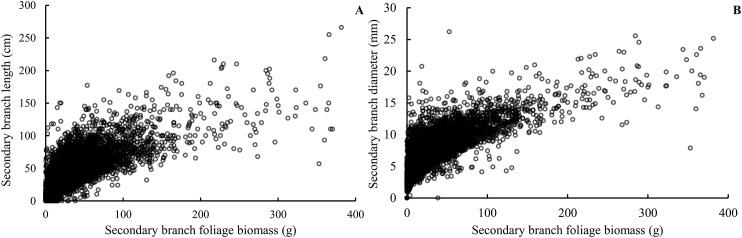
Relationship between the foliage biomass of the secondary branch and the secondary branch length **(A)** and the secondary branch diameter **(B)**.

Our study indicated that almost 82% of foliage biomass was grown from the secondary branches for the Korean pine. Secondary branches originating from the primary branch serve as the essentially important filling for the crown structure and thus affects crown function and photosynthetic product allocation (Kaitaniemi et al., 2020; Mäkinen and Hein, 2006; Kershaw et al., 2009). The sizes of the secondary branch, including length, diameter, and extension of the current-year shoot, were closely related to the foliage biomass distribution and growth of the primary branches (**Figure 10**) (Chen and Sumida, 2017). Quantitative and morphological characteristics of branches are important determinants of crown structure (Jensen and Long, 1983; Taugourdeau et al., 2012; Dong et al., 2016), since it directly influences light penetration as well as foliage distribution (Liu et al., 2023; Gao et al., 2023c). Therefore, understanding the secondary branch development could provide a deeper insight into understanding the crown function of individual trees (Goulet et al., 2000; Chen et al., 2010).

Growth space and light interception play an important role in determining the secondary branch distribution within the primary branch and entire crown (King et al., 1997; Chen and Sumida, 2017; MacFarlane and Kane, 2017). Branch length was much more sensitive to local light condition compared to branching frequency (Chen and Sumida, 2018). The reason might be that shoot length growth is superior to basal diameter growth. As a result, the secondary branch length has a limited range of variation originally, which will reduce the sensitivity of the diameter to variations. This was the main reason the relationship between the secondary branch length and the SDINC was stronger than the relationship between the secondary branch diameter and the SDINC. The corner rule points out that the larger the order of the branch, the smaller the size of the branches (Corner, 1949; Suzuki and Suzuki, 2009; Lauri, 2019). The results of our study showed that the relationship between the secondary branch length and diameter and the SDINC was weaker than the relationship between the primary branch length and diameter and the PDINC (**Figure 2**), which also demonstrated the corner rule. In addition, we found that the mean values of the secondary branch length showed a slight decreasing tendency with increasing tree age, partly because the growth of plants is accompanied by a decline in metabolism and energy (Niklas and Cobb, 2008; Niinemets, 2016). Because the length of the secondary branches served as an indicator to reflect the growth rate of the primary branches (Woollons et al., 2002; Zou et al., 2022), the length of the secondary branches decreased with increasing tree age indicating that younger trees benefit from more vigorous efforts than older trees.

The study of Lauri (2019) indicated that both internal and external factors have significant effects on the secondary branching structure. The age of the primary branch was an important factor affecting the secondary branch (Sattler et al., 2014). The age of the primary branch directly reflects the position of the branch on the trunk. Due to the different positions of the primary branch within the crown, the external factors affecting the development of the secondary branches were different (Umeki and Seino, 2003; Miao et al., 2021a). Consequently, secondary branches at the proximal part of the primary branches are predominantly influenced by light, whereas secondary branches at the distal part of the primary branches are completely in a shaded state and affected by competition. The relationship between the current increment of newly sprouted secondary branches and the age of primary branches was also studied in the present study. Sone et al. (2005) has demonstrated that the annual branch elongation has exhibited an impact on the thickening of the diameter. This was probably due to the fact that the upper branches of the tree will adopt a slender shape to carry more needles and improve light interception efficiency.

As the age of the primary branch increased, the mechanical support efficiency increased among perennial branches (Liu et al., 2021). Younger branches can get sufficient light, but have limitations on the transfer of nutrients. Older branches struggle to receive sufficient light and are also impacted by competition and inadequate nutrient distribution (Umeki and Seino, 2003; Chen and Sumida, 2018). Consequently, the annual increment of secondary branches shows a trend of either gradually decreasing or increasing and then decreasing. Thus, a detailed analysis was conducted on the relationship between the age of the primary branches and the growth of the newly sprouted secondary branches on the primary branches (Chavana-Bryant et al., 2017; Hayes et al., 2019). As for the effect of tree age on the secondary branch length and diameter, the resource allocation shift was probably the reasonable explanation for the result. Photosynthetic products were preferably allocated to primary growth in young trees to compete for light, and the secondary branch remained short (Koch et al., 2004). In comparison, more resources tended to allocate to the lateral crown expansion for the mature trees to obtain more competitive advantages (Gleason et al., 2016). As for the old trees, the photosynthetic products would shift to maintenance and reproduction by reducing the secondary branch elongation. In addition, the secondary branch within the different locations of the primary branch and crown were also affected by light competition and canopy structures. However, a comprehensive analysis for the interactions of each factor on the secondary branch size should be conducted in the future.

## 5 Conclusion

This study focused on the distribution pattern of secondary branch length and diameter within the primary branch and crown for planted Korean pine in northeast China. Almost 82% of foliage biomass grown from the secondary branches of Korean pine and foliage biomass from a specific secondary branch is closely related to the secondary branch length and diameter. Secondary branch length and diameter increased initially and subsequently decreased with increasing SDINC. The secondary branch structure develops as a result of both external impact and internal expression, with light and branch competition being the major external influences. Light interception and canopy structure in the forest were essentially important in shaping secondary branch sizes. Young trees, benefitting from the open canopy, developed longer secondary branches, whereas older trees experienced self-shading resulting in branch dieback and reduced branch length in the lower canopy. In addition, the comparison of the length and diameter of secondary branches shows that the variable pattern of diameter is less pronounced than that of length. Roeecp equation was the most effective base model for fitting secondary branch size with branch variables incorporated into the base model. The comprehensive secondary branch model showed satisfactory predictive ability in modeling secondary branch length and diameter for the Korean pine plantation in northeast China.

## Data Availability

The raw data supporting the conclusions of this article will be made available by the authors, without undue reservation.
